# Large-gap cascaded Moiré metasurfaces enabling switchable bright-field and phase-contrast imaging compatible with coherent and incoherent light

**DOI:** 10.1515/nanoph-2025-0494

**Published:** 2025-12-02

**Authors:** Yiyi Li, Wangzhe Zhou, Yuqing Zhang, Xiaoyan Huang, Yutai Chen, Man Yuan, Junbo Yang

**Affiliations:** College of Science, National University of Defense Technology, Changsha 410073, China; School of Physical Science and Technology, Southwest University, Chongqing 400715, China

**Keywords:** Moiré metasurfaces, optimized phase-iterative algorithm, bright-field imaging, multi-order edge-extraction imaging

## Abstract

Bright-field and phase-contrast imaging represent two of the most essential modes for target recognition and feature extraction, offering broad applicability in fields such as biomedicine and autonomous driving. In this work, we propose a cascaded Moiré metasurfaces system with a large interlayer spacing, which enables switchable bright-field and phase-contrast imaging at a wavelength of 532 nm by adjusting the illumination conditions between coherent and incoherent light sources. By employing an optimized phase-iterative algorithm, the stringent spacing requirement of conventional cascaded Moiré metasurfaces is relaxed from the subwavelength scale (∼100 nm) to beyond 1 mm, while maintaining robust imaging performance under spacing deviations of ±0.1 mm. Through controlled relative rotation of the two metasurfaces by an angle *θ*, the system dynamically switches between a focused solid Airy disk (*θ* = 0°) and vortex beams with tunable topological charges ranging from −5 to +5 (*θ* = ±20° to ±100°). The design achieves a focusing efficiency of 82 % and vortex beam purities up to 99 %. Owing to its versatile switching capability, the system supports multi-order edge extraction for both phase-type and amplitude-type objects, reaching a spatial frequency of 228 lp/mm. This approach overcomes the limitation of existing edge-detection metasurfaces, which operate only under either coherent or incoherent illumination. Our findings provide a new technical pathway toward compact, multifunctional, and integrated imaging devices.

## 1 Introduction

Image processing plays an indispensable role in a wide range of critical fields, including artificial intelligence, machine vision, biomedical imaging, and autonomous systems. Among various techniques, edge detection is one of the most widely used operations, serving as a crucial step for object detection and classification [1]. Conventionally, edge detection is implemented in the digital domain by processing captured images with suitable convolution kernels on computers. However, such digital approaches often suffer from high power consumption and limited processing speed, posing significant challenges for real-time computation and large-scale data processing. Moreover, the capability for massive parallel information handling remains restricted [2]. In contrast, optical image processing has emerged as a promising complementary approach to digital computing, offering high efficiency and low power consumption [3]. By performing edge detection in an all-optical manner during the image acquisition stage, this method provides near-light-speed processing, minimal energy loss, inherent parallelism, and high information capacity.

Conventional optical edge-detection techniques primarily rely on the Fourier transform effect of lenses. Typically, a 4f imaging system is employed, where a high-pass filter is inserted at the Fourier plane to enhance object contours by extracting higher-order information and suppressing irrelevant low-frequency components [4], [5]. This method is well suited for imaging intensity-absorbing samples (i.e., amplitude objects [6]). However, for highly transparent specimens (i.e., phase objects), contrast enhancement often requires staining, which interferes with cellular physiology and reduces cell viability. To address this limitation, alternative approaches have been developed by introducing tailored optical elements into the spatial filtering path to modulate the phase, amplitude, and spatial frequency at the sample plane [7]. Representative examples include Zernike phase-contrast imaging [8], [9] and differential interference contrast (DIC) imaging [10]. More recently, spiral phase contrast imaging has emerged as a promising edge-enhancement technique [11], [12], [13]. By introducing a *π* phase difference between the positive and negative spatial frequencies of the object, it enables the extraction of edge features from both amplitude- and phase-type samples. This method efficiently captures structural information and geometric features relevant to object boundaries, significantly reducing the amount of data to be processed. To date, spiral phase contrast imaging has been predominantly implemented using liquid-crystal-based phase plates and spatial light modulators, which provide dynamic control over the amplitude and phase of light. However, these implementations typically require multiple optical components, resulting in bulky setups with limited resolution and high costs [14], [15].

Metasurfaces, composed of subwavelength nanostructures, offer unprecedented control over the amplitude, phase, and polarization of light, holding great promise for the development of compact and high-performance imaging systems [16], [17], [18]. A variety of novel photonic devices based on metasurfaces have already been demonstrated, including metalenses, meta-holograms, ultrathin spectrometers, and optical filters [19], [20], [21], [22], [23], [24]. Recently, spiral phase contrast imaging implemented with metasurfaces has attracted significant attention due to its compact form factor, low cost, and multifunctional capabilities. However, most existing studies on metasurface-based edge detection have been carried out under the assumption of spatially coherent illumination. In practice, many imaging systems operate under spatially incoherent light, which poses additional challenges for real-world applications.

The introduction of Moiré metasurfaces [25], [26] has provided an alternative approach for realizing dynamically tunable and multifunctional spiral phase-contrast imaging systems, emerging as a promising route for edge detection under both coherent and incoherent illumination. Owing to their capability for continuous tuning, large adjustment range, and operational simplicity, Moiré metasurfaces have found applications in diverse areas such as biological microscopy [27], [28], terahertz 6G communications [29], and augmented reality displays [30]. However, most existing studies either neglect the spacing between cascaded metasurfaces or assume it to be half of the Talbot distance. Under this idealized assumption, the phase profiles of the two layers can be directly superimposed during design. In practice, Moiré metasurfaces with wavelength-scale interlayer spacing face severe challenges in large-scale implementation due to stringent requirements on layer alignment and motion control. Larger interlayer distances further degrade imaging quality, resulting in blurred images [31]. Although special phase design methods have been proposed to extend the gap to several wavelengths [32], [33], the achievable spacing in the visible regime remains limited to the micrometer scale. Approaches based on geometrical ray-tracing have also been investigated to enlarge the interlayer spacing [34], but these methods neglect the wave nature of light.

To address these challenges, we propose an iterative algorithm based on angular spectrum propagation theory, enabling high-precision phase retrieval for complex optical fields. After optimization, this algorithm relaxes the stringent spacing requirements of conventional cascaded Moiré metasurfaces from the sub-100-nm scale to beyond 1 mm, while maintaining stable imaging performance under interlayer spacing deviations of ±0.1 mm. Building on this approach, we design a Moiré metasurfaces device that integrates the phase profiles of a spiral phase plate and a lens, allowing flexible and active switching between a focused Airy disk and vortex beams via rotation of the metasurfaces. The results show that relative rotation of the two-layer device not only produces a focused solid Airy disk but also generates vortex beams with topological charges tunable continuously from −5 to +5. The system is compatible with both coherent and incoherent illumination for edge detection. This compact, high-resolution, and dynamically tunable spiral phase-contrast imaging platform holds significant potential for practical applications in biomedicine [35], object recognition, and microscopic imaging.

Table 1 compares the present study with previously reported dual-mode imaging metasurface systems. Previous studies have almost exclusively employed static metasurfaces capable of performing only a single function or processing task, limiting their applicability and impact in complex systems. In 2022, a study demonstrated first-order spiral phase contrast imaging [36], but the system lacked tunability and bright-field imaging capability. Subsequent work combined deflecting phase elements to achieve simultaneous bright-field and first-order spiral phase contrast imaging [37]; however, the two imaging modes suffered from crosstalk and were vulnerable to zeroth-order diffraction. Subsequent studies have employed various control mechanisms to achieve switching between different imaging modes. By toggling the spin state of the incident light (left- or right-handed circular polarization), dual-functional imaging has been realized using either a single spiral metasurface [38], [39] or a 4f optical system [40]. Mie-resonant metasurfaces have also been utilized to achieve dual-mode imaging with different contrast levels by switching the polarization state of the incident light [41]. In addition, thermal control of phase-change materials has been exploited to develop reconfigurable metasurfaces capable of performing both bright-field and edge-enhanced imaging [42], [43]. However, these imaging demonstrations remain limited to bright-field and first-order spiral phase-contrast imaging. Moreover, most of these studies were conducted under coherent illumination, whereas practical imaging scenarios often require operation under ambient incoherent light. Consequently, several approaches have been explored to enable edge detection or image differentiation under incoherent illumination, such as multilayer films [44], photonic crystals [45], and spiral metalenses [46]. However, due to the inherent limitations of the incoherent point spread function, these methods often rely on wavelength multiplexing or deflection multiplexing to achieve effective differentiation [47]. Recently, Swartz et al. employed an inverse-optimized hybrid system combining a metasurface and a refractive lens to realize bipolar Laplacian kernels, enabling edge detection of broadband incoherent thermal radiation in the long-wave infrared regime [48]. Considering the complexity of real-world illumination environments, it is therefore desirable to develop all-optical two-dimensional imaging devices that can operate under both coherent and incoherent illumination, thereby extending the capability boundary of optical image processing. The 4f imaging system has been shown to perform spatial differentiation and edge detection under both coherent and incoherent illumination [40], [49]. However, the switching between bright-field and edge-enhanced imaging in these systems is typically achieved through polarization multiplexing or by inserting and removing the metasurface within the 4f configuration. To further simplify the system architecture, reduce the complexity of dual-mode control, and enhance multifunctionality, this work proposes a cascaded Moiré metasurfaces system capable of dynamically switching between bright-field and spiral phase-contrast imaging under both coherent and incoherent illumination through relative rotation, and further extending the functionality to multi-order edge detection. Different edge orders can be interpreted as selective control over specific spatial frequencies, providing a potential approach for feature analysis in the high-dimensional orbital angular momentum (OAM) spectral space [50], [51], with prospective applications in facial recognition, optical character recognition, and artificial intelligence vision systems. Moreover, recent studies have demonstrated the remarkable multifunctional potential of Moiré metasurfaces, integrating functions such as edge extraction and quantitative phase imaging [52] or tunable focusing and zooming [53] through relative rotation of cascaded metasurfaces. Furthermore, existing studies on Moiré metasurfaces typically limit the interlayer spacing of cascaded metasurfaces to the micrometer scale [52], [53], [54], requiring stringent control for practical implementation. Here, the interlayer spacing is extended to the millimeter scale, while maintaining stable imaging performance under spacing deviations of ±0.1 mm. Taken together, this work focuses on designing a Moiré metasurfaces system capable of operating at large interlayer spacing, integrating bright-field imaging with multi-order spiral phase contrast imaging, offering dynamic tunability and compatibility with diverse imaging scenarios. Such a system is expected to provide significant processing advantages and broaden the scope of practical applications. It is worth noting that in Table 1, “dual-mode” refers to the ability of a metasurface system to perform both bright-field and spiral phase-contrast imaging either simultaneously or through mode switching. “Tunability” denotes the capacity to dynamically switch between the two imaging modes via external control, such as polarization, rotation, phase change, or mechanical insertion/removal of optical elements.

**Table 1:** Comparison of the present study with previously reported dual-mode imaging metasurface systems.

Ref	Type	Illumination	Bright-field imaging	Spiral phase order	Dual-mode imaging	Tunability	Interlayer spacing
[36]	Spiral	Coherent	False	1	False	False	–
[37]	Spiral	Coherent	True	1	True	False	–
[38], [39]	Spiral	Coherent	True	1	True	Polarization	–
[40]	4f	Coherent & incoherent	True	1	True	Polarization	–
[41]	Mie resonances	Coherent	True	1	True	Polarization	–
[42], [43]	Single	Coherent	True	1	True	Phase change	–
[46]	Spiral	Incoherent	True	1	True	False	–
[48]	Hybrid	Incoherent	True	1	True	Polarization	–
[49]	4f	Coherent & incoherent	True	1	True	Insertion/removal	–
[52]	Moiré	Coherent	False	1	False	False	<5 μm
[53]	Moiré	Incoherent	True	1	False	False	<35 μm
[54]	Moiré	Coherent	False	−3 to +3	False	False	0.25 μm
**This work**	Moiré	Coherent & incoherent	True	−5 to +5	True	Rotation	0.9–1.1 mm

## 2 Principle and design method

**Figure 1:**
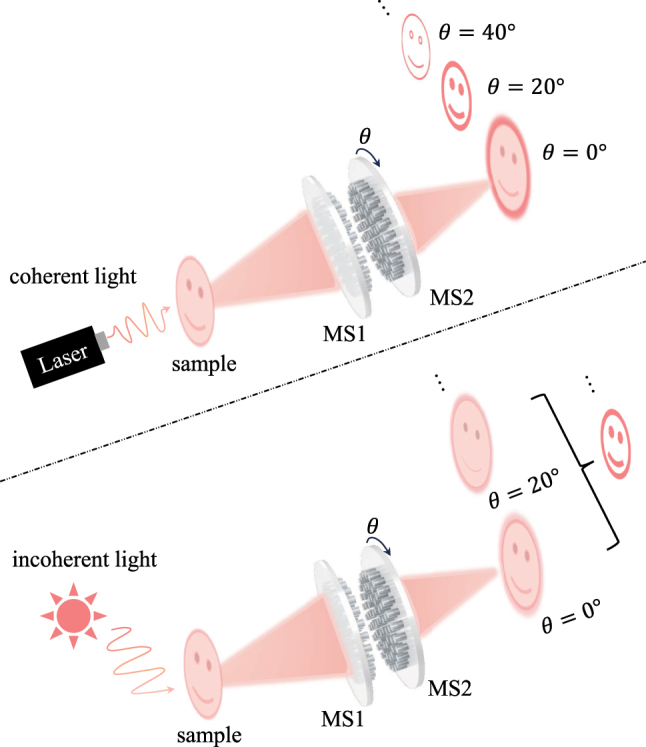
Schematic of the Moiré metasurfaces device for edge detection under both coherent and incoherent illumination.

In this work, we propose a cascaded Moiré metasurfaces capable of flexibly switching between bright-field and phase-contrast imaging, as illustrated in Figure 1. The system consists of two metasurfaces, MS1 and MS2, which are axially aligned and placed face to face. Imaging of target objects can be achieved under either coherent illumination (e.g., laser sources) or incoherent illumination (e.g., natural light). By adjusting the illumination conditions, the system enables switching between coherent and incoherent illumination configurations. When MS2 is rotated relative to MS1 by an angle *θ*, the Fourier spectrum of the system is correspondingly modified, generating distinct filtering responses. As a result, the cascaded structure enables dynamic switching between bright-field imaging and multi-order edge-enhanced imaging, thereby extracting multi-level structural information of the object.

### 2.1 Dynamically tunable topological charge

The fundamental principle for constructing the Moiré metasurfaces device is described as follows. By cascading two metasurfaces with carefully engineered phase responses, Moiré patterns are generated through their relative rotation, which dynamically modulates the wavefront of the transmitted light. To realize dynamic control of vortex beams, the phase profiles of the two cascaded metasurfaces in polar coordinates are defined as $\varphi_{1}\left(r,\varphi \right)=-\varphi_{2}\left(r,\varphi \right)=\varphi^{2}$ where *φ* is the azimuthal angle varying from 0 to 2*π*, and *r* denotes the radial coordinate. When one metasurface is rotated relative to the other by an angle *θ*, the resulting total phase distribution can be expressed as:

(1) $$\begin{array}{c} \varphi_{\text{joint}}=\varphi_{1}\left(r,\varphi \right)+\varphi_{2}\left(r,\varphi -\theta \right)=2\theta \varphi -\theta^{2} \end{array}$$

Due to the intrinsic periodicity of the rotation in Moiré metasurfaces devices, a sector-shaped effect inevitably arises. To address this issue, phase quantization and compensation are required [55]. Accordingly, the phase distributions of the two metasurfaces, as well as their cascaded phase, can be expressed as:

(2) $$\begin{array}{ll} \varphi_{1}\left(r,m\right) & =\frac{m^{2}}{N}\pi \\ \varphi_{2}\left(r,m\right) & =-\frac{m^{2}}{N}\pi -\frac{2\pi }{\lambda_{0}}\left(f-\sqrt{r^{2}+f^{2}}\right)\\ \varphi_{\text{joint}} & =n\cdot \text{round}\left(\frac{\varphi }{\Delta \varphi }\right)\cdot \Delta \varphi -\frac{\pi }{N}n^{2}\\ & \;-\frac{2\pi }{\lambda_{0}}\left(f-\sqrt{r^{2}+f^{2}}\right) \end{array}$$

Here, *m* is an integer defined as $m=\text{round}\left(\varphi /\Delta \varphi \right)$ . *N* is typically chosen as an even number, given by *N* = 2*π*/Δ*φ*, indicating that the angular range of the metalens (0−2*π*) is divided into *N* small sectors of size Δ*φ*. *λ*_0_ denotes the target free-space operating wavelength, $\left(r,\theta \right)$ are the polar coordinates on the metasurface plane, and *f* is the focal length of the metalens. To ensure an integer topological charge, the rotation angle *θ* must be an integer multiple of Δ*φ*, expressed as *θ* = *n*Δ*φ*, where *n* is an integer satisfying −*N* ≤ *n* ≤ *N*. Since vortex beams inherently exhibit divergence, a hyperbolic phase is introduced to focus the vortex beam. Compared with the conventional spiral phase $\varphi_{\text{spiral}}\left(r,\varphi \right)=l\varphi$ , the topological charge *l* can be modulated by the sector number *N* and the relative rotation *θ* [56], which can be expressed as:

(3) $$\begin{array}{c} \begin{array}{c} l=n=N\frac{\theta }{360{}^{\circ}} \end{array} \end{array}$$

**Figure 2:**
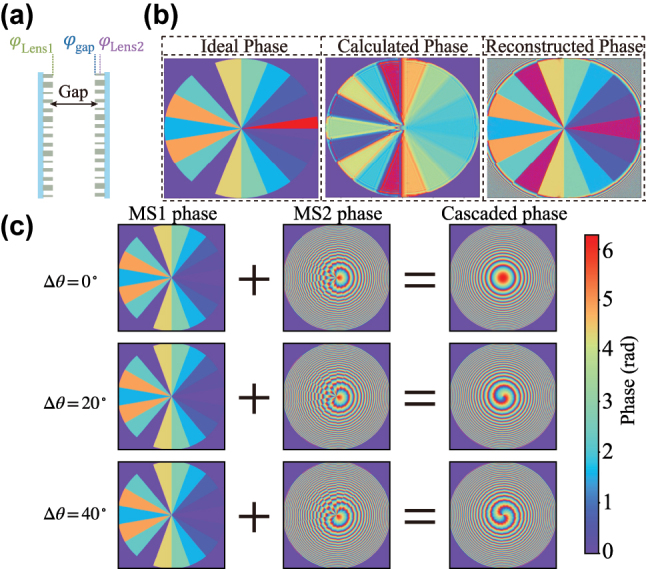
System configuration and phase profiles of the cascaded Moiré metasurfaces. (a) Schematic of the relative alignment of the two metasurfaces. (b) From left to right: the ideal phase profile of the first metasurface, the phase distribution obtained through the iterative phase retrieval algorithm, and the propagated phase distribution before reaching the second metasurface. (c) Phase distributions of the two metasurfaces at different relative rotation angles and their corresponding cascaded phase profiles.

Thus, the proposed Moiré metasurfaces device dynamically controls the topological charge of vortex beams by cascading two metasurfaces with specially engineered phase distributions and rotating them relative to each other. As illustrated in Figure 2(c), when the relative rotation angle is zero, the cascaded metasurfaces exhibit the phase profile of a hyperbolic lens, thereby functioning as a focusing element. Once the second metasurface is rotated, the overall phase distribution transforms into a spiral phase. With increasing rotation angle, the order of the spiral phase correspondingly increases, enabling dynamic modulation of the vortex beam order.

### 2.2 Iterative phase algorithm

**Figure 3:**
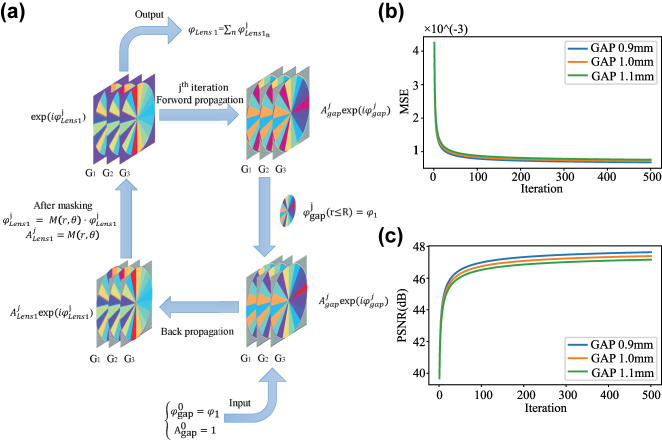
Iterative phase optimization across different interlayer spacings. (a) Flowchart of the iterative phase algorithm across multiple interlayer spacings. (b) Corresponding mean squared error (MSE) results. (c) Corresponding peak signal-to-noise ratio (PSNR) results.

As illustrated in Figure 2(a), when the two metasurfaces are placed sufficiently close such that diffraction across the interlayer gap can be neglected, the phase distributions in Eq. (2) can be directly added. However, when the interlayer spacing increases, the phase *φ*_lens1_ of the first metasurface diffracts before reaching the second metasurface and deviates from the intended phase *φ*_1_. To address this, we propose a novel phase design paradigm based on the angular spectrum propagation (ASP) theory, as shown in Figure 3(a), allowing MS1 and MS2 to maintain near-ideal imaging performance even at large interlayer spacings. The ASP method strictly satisfies the Helmholtz equation, providing highly accurate and reliable diffraction calculations between input and output planes during the iterative procedure [7]. Meanwhile, employing the scalar diffraction approximation significantly reduces computational cost and hardware resource requirements, making it well-suited for evaluating phase design accuracy. Ideally, the phase distribution arriving at the front surface of MS2 is the target phase *φ*_1_ in Eq. (2), which serves as the iteration target. That is, after the j-th iteration, we aim for $\varphi_{\text{gap}}^{j}\approx \varphi_{1}$ , with the initial input set as $\varphi_{\text{gap}}^{0}=\varphi_{1}$ . Since the optical field has a complex amplitude, the amplitude must also be considered. The initial amplitude is set as $A_{\text{gap}}^{0}=1$ . The complex amplitude is then back-propagated over the prescribed gap *G* to MS1, yielding $\varphi_{\text{Lens}1}^{0}$ and $A_{\text{Lens}1}^{0}$ . Due to the finite aperture of the actual metasurface, components outside the aperture are set to zero: $\varphi_{\text{Lens}1}^{0}=\varphi_{\text{Lens}1}^{0}\cdot M\left(r,\theta \right)$ , where $M\left(r,\theta \right)$ is a mask function equal to 1 within the metasurface radius *R* and 0 elsewhere. In this study, the metasurfaces are treated as pure phase devices, so the amplitude is also adjusted: $A_{\text{Lens}1}^{0}=M\left(r,\theta \right)$ .

The processed complex amplitude, $A_{\text{Lens}1}^{0}\text{exp}\left(i\varphi_{\text{Lens}}^{0}\right)$ , is then forward-propagated to obtain the complex field at the front of the second metasurface, $A_{\text{gap}}^{0}\text{exp}\left(i\varphi_{\text{gap}}^{0}\right)$ . The resulting phase $\varphi_{\text{gap}}^{0}$ is compared with the ideal phase distribution of the first metasurface, *φ*_1_(*r*, *m*), and the corresponding mean squared error (MSE) and peak signal-to-noise ratio (PSNR) are computed. The definitions of MSE and PSNR are given by:

(4) $$\begin{array}{c} \begin{array}{c} \text{MSE}=\frac{1}{ab}\overset{a}{\underset{i=1}{\sum }}\overset{b}{\underset{k=1}{\sum }}{\left(\varphi_{\text{gap}}^{j}-\varphi_{1}\right)}^{2}\\ \text{PSNR}=10*\log_{10}\left(\frac{\text{MA}{\text{X}}^{2}}{\text{MSE}}\right) \end{array} \end{array}$$

Here, *φ*_1_ denotes the ideal phase distribution of the first metasurface, as given in Eq. (2). *a* and *b* represent the number of sampling points along the two phase matrices, *j* is the iteration number, and MAX denotes the maximum value among the matrix elements.

If the convergence criterion is not satisfied, the phase distribution $\varphi_{\text{gap}}^{0}$ within the aperture is replaced with the corresponding values from *φ*_1_, while the values outside the aperture remain unchanged. The amplitude $A_{\text{gap}}^{0}$ is kept unchanged. The updated complex field, $A_{\text{gap}}^{0}\text{exp}\left(i\varphi_{\text{gap}}^{0}\right)$ , is then back-propagated from the front surface of the second metasurface to the first metasurface. This procedure is repeated iteratively until either the convergence criterion is met or the maximum number of iterations is reached.

However, unavoidable variations in the interlayer spacing may occur during adjustment. To enhance the robustness of the device, we assume that its performance remains stable over a range of spacings $G=\left[G_{1},G_{2},\ldots G_{n}\right]$ , where *n* denotes the number of discrete spacing values. For each spacing *G*_*n*_, the phase distribution of MS1, $\varphi_{\text{Lens}1_{n}}$ , is calculated using the iterative phase algorithm, resulting in $\varphi_{\text{Lens}1}=\left[\varphi_{\text{Lens}1_{1}},\varphi_{\text{Lens}1_{2}}\ldots \varphi_{\text{Lens}1_{n}}\right]$ . Using a holographic design approach [57], the computed phase distributions for all spacings are then superposed to obtain the final phase distribution of the first metasurface: $\varphi_{\text{Lens}1}=\sum \varphi_{\text{Lens}1_{n}}$ .

## 3 Results and discussion

### 3.1 Phase distribution after iterative optimization

In this study, the focal length of the metasurface device was set to *f* = 1 mm, with an incident wavelength of 532 nm. The radii of both metasurfaces were *R* = 0.5 mm, corresponding to a numerical aperture (NA) of 0.5. Due to the quantization of the spiral phase distribution on the Moiré metasurfaces, fractional segments may affect imaging performance [40]. To progressively approach ideal spiral phase-contrast imaging, the spiral phase was divided into *N* = 18 sectors, each with an angular interval of Δ*φ* = 20°. The rotation angle *θ* was defined in the range $\left[-180{}^{\circ},180{}^{\circ}\right]$ , with positive values indicating clockwise rotation and negative values indicating counterclockwise rotation. Moreover, *θ* was restricted to integer multiples of Δ*φ*. Assuming an interlayer spacing of 1 mm, and to ensure robust performance under small deviations, the spacing was varied within the range 0.9 mm–1.1 mm, divided into three discrete values: *G*_1_ = 0.9 mm, *G*_2_ = 1.0 mm, and *G*_3_ = 1.1 mm. These parameters were input into the iterative phase algorithm illustrated in Figure 3(a), and the corresponding MSE and PSNR were calculated for each spacing, as shown in Figure 3(b) and (c). Over 500 iteration cycles, the MSE for all three spacings gradually decreased, achieving an overall reduction of 82.3 %. Similarly, the PSNR increased by 7.51 dB with iteration number. Smaller interlayer spacings corresponded to lower MSE and higher PSNR values. The final phase distribution of the first metasurface is shown in Figure 2(b) as the *calculated phase*, and the propagated phase after 1 mm, just before reaching the second metasurface, is shown as the *reconstructed phase*. Compared with the ideal phase, the final MSE reached 0.07 %, and the PSNR approached 48 dB, indicating excellent phase fidelity. These results demonstrate that the iterative algorithm effectively mitigates phase distortions caused by increased interlayer spacing, while maintaining stable phase reconstruction across the specified spacing range.

**Figure 4:**
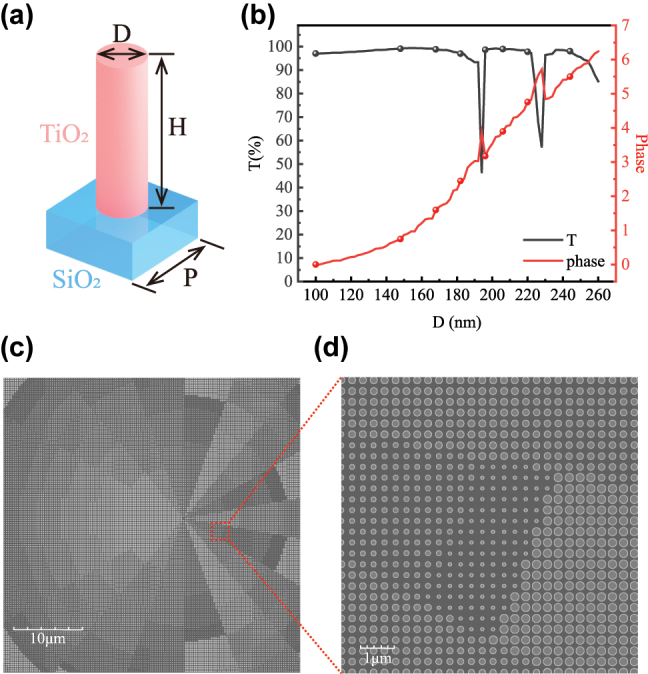
Unit cell design and phase discretization of the second metasurface. (a) Schematic of the unit cell structure. (b) Transmission amplitude and phase response of TiO_2_ nanopillars with height *H* = 700 nm and period *P* = 320 nm as a function of the diameter *D*. (c, d) Distribution of meta-atoms in the central region of MS2 based on the optimized discretized phase profile.

After obtaining the phase distribution of the metasurface, a set of meta-atoms covering the full 0–2*π* phase range was selected for discretized design. As illustrated in Figure 4(a), polarization-insensitive cylindrical nanopillars were chosen as the basic building blocks. The nanopillars were fabricated from high-refractive-index TiO_2_ and placed on a SiO_2_ substrate, both of which offer low cost and compatibility with scalable nanofabrication processes [58]. In the design, the nanopillars were arranged in a subwavelength square lattice with a period of *P* = 320 nm and a height of *H* = 700 nm. Finite-difference time-domain (FDTD) simulations were performed to determine the transmission amplitude and phase response for diameters D ranging from 100 nm to 260 nm. From these results, eight discrete geometries were selected to uniformly cover the full 0–2*π* phase range, as shown in Figure 4(b), with an average transmission efficiency of 98 %. The design and fabrication of the corresponding Moiré metasurfaces have been extensively validated through various simulations and theoretical models, and the structural dimensions and materials are fully compatible with current nanofabrication technologies [30], [52], [59]. Detailed fabrication procedures are provided in Supplementary Information S1.

### 3.2 Generation of vortex beams with tunable topological charge under varying spacings and rotation angles

**Figure 5:**
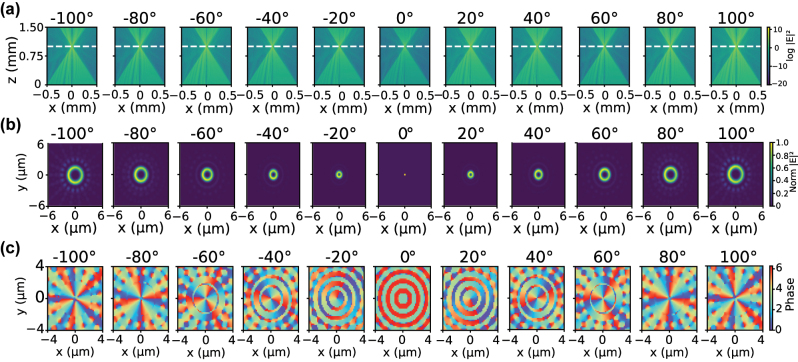
Generation of vortex beams with tunable topological charge using the Moiré metasurfaces device, with the relative rotation angle between the two metasurfaces varied from −100° in steps of 100°. (a) Normalized field distribution along the propagation direction in the *x*–*z* plane; the white dashed line indicates the focal plane. (b) Normalized intensity distribution in the *x*–*y* plane at the focal plane. (c) Phase distribution in the *x*–*y* plane at the focal plane.

To verify the feasibility of the proposed scheme, the *calculated phase* obtained from the iterative algorithm was used as the phase distribution of the first metasurface, while $\varphi_{2}\left(r,m\right)$ from Eq. (2) was assigned to the second metasurface. The phase and amplitude responses of the unit structure shown in Figure 4(b) are employed, and the meta-atoms are arranged according to the respective phase distributions of each metasurface. Figure 4(c) and (d) illustrate the distribution of meta-atoms in the central region of MS2 based on the optimized discretized phase profile. The interlayer spacing was set to 1 mm, and the incident wavelength was 532 nm. The transmission of the cascaded Moiré metasurfaces device was simulated using the plane-wave angular spectrum diffraction method. Simulations were performed for various rotation angles *θ*, as shown in Figure 5, with *θ* ranging from −100° to 100° in steps of 20°. It can be clearly observed that the focal length remains 1 mm for all rotation angles, consistent with the design. When the relative rotation between the two metasurfaces is 0°, the focal-plane spot corresponds to a solid Airy disk, carrying no spiral phase; the amplitude distribution exhibits a bright central point, while the phase distribution forms concentric rings with constant phase. As *θ* increases, the intensity distribution gradually evolves into a doughnut-shaped ring, with the ring radius increasing with *θ*. Simultaneously, the corresponding phase exhibits multiple 2*π* spiral cycles, with the topological charge *l* following the relation given by Eq. (3), ranging from −5 to 5. Clockwise or counterclockwise rotations of the same magnitude produce ring-shaped intensity distributions with identical radii but opposite handedness. The tunable range of the topological charge is determined by the relative rotation angle *θ* and the number of angular sectors *N*.

**Figure 6:**
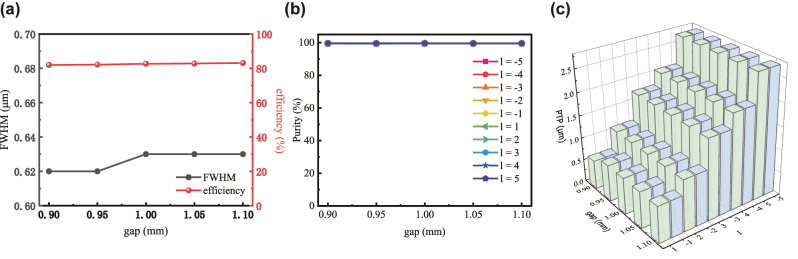
Focusing analysis of the cascaded Moiré metasurfaces for different interlayer spacings and relative rotation angles. (a) Focusing efficiency and full width at half maximum (FWHM) of the focused spot at a rotation angle of 0° for various spacings. (b) Modal purity of vortex beams corresponding to the topological charges in Eq. (3) under different rotation angles and interlayer spacings. (c) Peak-to-peak (PTP) diameters of vortex beams for different interlayer spacings and relative rotation angles.

To verify the stability of the device’s focusing performance within the interlayer spacing range of 0.9 mm–1.1 mm, five spacings were selected with a step of 0.05 mm. For each spacing, the relative rotation of the metasurfaces was varied from −100° to 100° in steps of 20°, corresponding to topological charges of −5, −4, −3, −2, −1, 1, 2, 3, 4, 5. When the rotation angle is 0°, no spiral phase is introduced, and the size of the focused spot is evaluated using the full width at half maximum (FWHM). As shown in Figure 6(a), the FWHM remains largely consistent across different spacings: 0.62 μm for 0.9 mm and 0.95 mm, and slightly increasing to 0.63 μm for spacings above 1.0 mm. The theoretical diffraction-limited FWHM is *λ*/2NA = 0.53 μm, indicating that the system operates near the diffraction limit. In addition, the focusing efficiency is defined as the fraction of energy within a circle of radius three times the FWHM at the focal plane:

(5) $$\begin{array}{c} \eta \left(\rho_{0}\right)=\frac{\overset{\rho_{0}}{\underset{0}{\int }}\overset{2\pi }{\underset{0}{\int }}I\left(\rho ,\varphi \right)\rho d\rho d\varphi }{\overset{\infty }{\underset{0}{\int }}\overset{2\pi }{\underset{0}{\int }}I\left(\rho ,\varphi \right)\rho d\rho d\varphi } \end{array}$$

Here, $I\left(\rho ,\varphi \right)$ denotes the intensity distribution at the focal plane, and *ρ*_0_ = 3FWHM/2. For large interlayer spacings, even when the relative spacing exhibits a tolerance of ±0.1 mm, the device maintains an average focusing efficiency of 82 %, with no significant change in the focal spot profile. This demonstrates that the phase-iteration algorithm effectively preserves bright-field imaging quality under large interlayer separations.

When the relative rotation is nonzero, the system generates vortex beams with different topological charges. To quantitatively evaluate the quality of the output vortex beams, the modal purity of the multi-mode vortex beam is introduced [60]. The power of the target orbital angular momentum (OAM) mode is first extracted from the field distribution:

(6) $$\begin{array}{c} p_{l}=\frac{1}{2\pi }\overset{\infty }{\underset{0}{\int }}{\left|\overset{2\pi }{\underset{0}{\int }}u\left(x,y\right)\cdot \exp\left(-jl\varphi \right)\text{d}\varphi \right|}^{2}r\text{d}r \end{array}$$

Here, $u\left(x,y\right)$ represents the complex amplitude of the electric field. The modal purity *P*_*l*_ of the target mode is defined as the fraction of power carried by the desired OAM mode relative to the total power of the generated vortex beam, i.e.,

(7) $$\begin{array}{c} P_{l}=\frac{p_{l}}{\overset{+\infty }{\underset{q=-\infty }{\sum }}p_{q}} \end{array}$$

The vortex beams corresponding to the topological charges in Eq. (3) were taken as the target modes, and their modal purities were extracted for different interlayer spacings, as shown in Figure 6(b). It is observed that the cascaded Moiré metasurfaces model designed via the phase-iteration algorithm maintains high modal purities of up to 99 % for vortex beams of various orders across the large spacing range of 0.9 mm–1.1 mm. This indicates that the phase-iteration algorithm effectively preserves the performance of the system over a wide spacing range, ensuring that the quality and focusing performance of vortex beams of different orders remain uncompromised. Furthermore, the peak-to-peak (PTP) diameters of vortex beams with different topological charges were evaluated under varying spacings and relative rotation angles, as shown in Figure 6(c). For a rotation angle of 20°, the first-order vortex beam exhibits a PTP of 0.64 μm. When the rotation angle increases to 100°, the fifth-order vortex beam has a PTP of 2.27 μm. The PTP of the first-order vortex beam closely matches the FWHM of the hyperbolic metasurface lens [36]. Vortex beams with topological charges of equal magnitude but opposite sign exhibit identical PTPs. At a fixed spacing, increasing the relative rotation angle results in higher topological charges and correspondingly larger PTPs. Smaller PTP values correspond to higher achievable resolution [36]. In addition, for different interlayer spacings, the PTPs of vortex beams of various orders remain nearly unchanged, further confirming the strong robustness of the system across the large spacing range. A detailed tolerance analysis is presented in Supplementary Sections S2 and S3. Specifically, the effects of lateral misalignment along the *x*-direction and longitudinal misalignment along the *y*-direction between the two metasurfaces on the imaging performance are analyzed, together with the influence of rotational accuracy between the two layers. The combined results demonstrate that the proposed Moiré metasurfaces system exhibits strong robustness against both positional and angular fabrication tolerances.

### 3.3 Edge extraction under coherent illumination

To demonstrate the cascaded Moiré metasurfaces’ capability for switching between bright-field and spiral phase-contrast imaging, we performed imaging simulations using the system’s point spread function (PSF) discussed above. In an ideal coherent imaging system, the electric field of the resulting image can be expressed as the convolution of the object’s electric field with the complex-amplitude *psf* of the system, i.e.,

(8) $$\begin{array}{c} U_{o}\left(x_{o},y_{o}\right)=U_{i}\left(x_{i},y_{i}\right)*psf \end{array}$$

**Figure 7:**
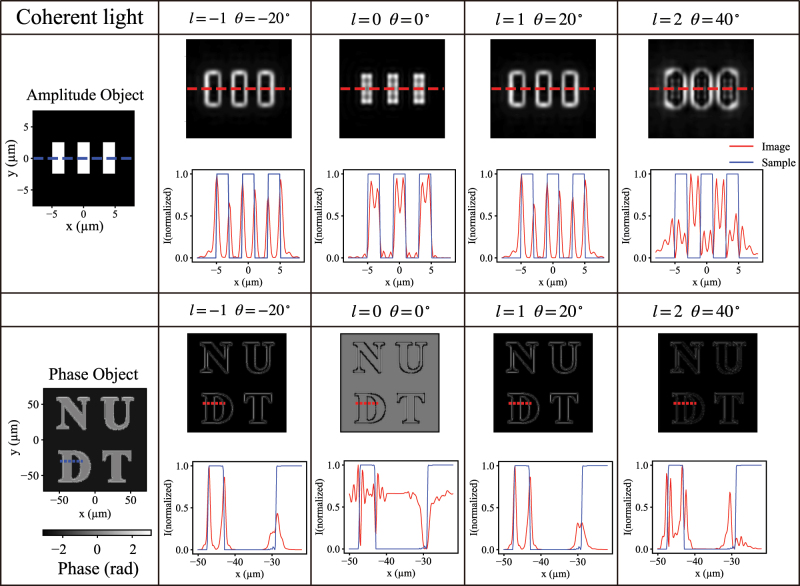
Edge-detection results of the cascaded Moiré metasurfaces for amplitude and phase objects under coherent illumination, with relative rotation angles of −20°, 0°, 20°, and 40°. Red lines indicate the imaging results, and blue lines represent the actual object distribution.

Here, $U_{i}\left(x_{i},y_{i}\right)$ denotes the complex amplitude of the input electric field, $U_{o}\left(x_{o},y_{o}\right)$ denotes that of the output electric field, ∗ denotes the convolution operator, and *psf* represents the complex-amplitude point spread function numerically calculated using the plane-wave angular spectrum method. Figure 7 presents the bright-field and multi-order spiral phase-contrast imaging results for both amplitude and phase objects under coherent illumination. For testing, we selected three rectangular slits from Group 7, Element 6 of the USAF 1951 resolution target as amplitude objects. When the relative rotation angle between the metasurfaces is 0°, the system functions as a low-pass filter, producing bright-field images at the image plane. Although intensity non-uniformity arises from the phase superposition corresponding to different interlayer gaps in the iterative algorithm, the three rectangular slits are still clearly resolved. Upon rotating the metasurfaces, the cascaded structure directly performs edge extraction. This occurs because the system’s PSF now carries a spiral phase, introducing a *π* phase difference at opposite azimuths. Integration over uniform regions of the input field leads to destructive interference and a dark background, while regions with phase and amplitude gradients (edges) produce incomplete interference cancellation, thereby enhancing the edge signals. When rotated clockwise by 20°, the system generates high-intensity single-peak enhancement at the boundaries of amplitude objects, achieving isotropic global edge detection. The spiral phase filtering effectively acts as a two-dimensional spatial derivative of the incident field. For a counterclockwise rotation of 20°, a similar edge-enhancement effect occurs, with the sign of the topological charge mainly determining the directionality of edge enhancement, resulting in a mirror-symmetric profile.

As the rotation angle increases, the corresponding topological charge also increases. The inner radius of the ring-shaped intensity distribution enlarges, acting as a high-pass filter that suppresses low- and mid-frequency components, further affecting edge enhancement. At a relative rotation of 40°, edges appear at the minima of the intensity signal, with a double-peak structure on either side. Here, the topological charge is *l* = 2, and the doubled spiral phase means that straight edges no longer satisfy the phase condition corresponding to *l* = 1, reducing the sharpness of right-angle edges. Notably, the multi-order spiral phase-filtered edge images differ from the multiple-edge effects observed in Bessel vortex beams [61], as the PSF output does not follow a power-law relationship with spatial wave vectors [62]. Consequently, the output field does not exhibit a simple differential effect. These results demonstrate that edge detection with different vortex orders essentially constitutes a spatial filtering operation that selectively modulates spatial frequencies. In practical applications, first-order vortex beams are suitable for extracting edge intensity, while second-order beams can emphasize fine details and contours. However, as more low-frequency components are filtered out, increased noise and artifacts may appear in the resulting images.

To further evaluate the edge-detection capability of the cascaded Moiré metasurfaces for phase objects under coherent illumination, we consider a pure-phase object represented as $U_{\text{in}}\left(x,y\right)=\exp\left[i\phi \left(x,y\right)\right]$ . Here, the object consists of the letters “NUDT” embedded in a background with a phase difference of *π*. The corresponding imaging results are shown in Figure 7. When the metasurfaces are not rotated, the structural information is entirely masked by the low-frequency background. Upon rotation, vortex beams with equal but opposite topological charges produce similar edge-enhancement effects, generating single-peak enhancement at the edges. For a relative rotation angle of 40°, edges appear at the intensity minima between the double peaks. Higher-order filtering channels further enhance curved and angular contours, allowing clear visualization of the spatial structure and features of the optical field. However, the higher-order filtering channels not only strengthen these contours but also modify the apparent image scale and feature width. This effect arises because increasing the topological charge expands the radius of the vortex-shaped PSF, which effectively suppresses a larger portion of the low-spatial-frequency content while preserving only higher-frequency components near the object edges. Such apparent magnification changes are therefore not true geometric magnification but rather a frequency-domain bias introduced by high-order filtering. The phenomenon can be more clearly verified by using the edge extraction results of a single slit under different frequencies and orders of topological loads in the Supplementary Material S7 Section.

In Supplementary Section S5, we perform global normalization of the edge-enhancement results for orders *l* = 1–5. When *l* = 2, the peak intensity is only half of that for *l* = 1, and it continues to decrease as the topological charge increases, making the edges progressively less visible. Even in the one-dimensional cross-sections of Figure 7, where normalization is applied, the higher-order edges remain nearly imperceptible. This behavior arises because the FWHM associated with the edge response becomes smaller as *l* increases, resulting in narrower visible linewidths. As shown in Figure 5, the donut radius of the PSF increases with topological charge, meaning that a larger portion of the mid- and low-frequency components is filtered out. Consequently, the useful edge information is further suppressed while noise is amplified, weakening the edge content that is actually transferred to the image plane. This view is also verified in Supplementary Material S7. It is therefore evident that edge detection cannot be improved simply by filtering out more spatial-frequency components. Once the useful edge information is excessively attenuated, the relative contribution of noise becomes dominant, leading to a reduction in the effective contrast and a decline in the perceptual visibility of the edges. To enhance the visibility of high-order edges, one feasible approach is to increase the effective numerical aperture (NA) of the system, thereby allowing more high-frequency components to fall within the detectable angular range. Alternatively, phase or amplitude compensation strategies can be incorporated into the metasurface design to preserve more energy within the critical spatial-frequency band for higher-order vortex modes.

For this typical imaging mode, the resolution test uses the smallest line pairs of Group 7, Element 6 of the USAF 1951 target, corresponding to a spatial frequency of 228 lp/mm and a line width of 2.19 μm. All edge-detection operations are performed in parallel without additional power consumption or image-processing algorithms, highlighting the potential of this system for advanced imaging and optical computing applications.

### 3.4 Edge extraction under incoherent illumination

For an incoherent imaging system, the captured image can be expressed as the convolution of the object’s intensity with the system’s intensity point spread function (PSF), i.e.,

$$\begin{array}{c} I_{\text{out}}=\text{PSF}*I_{\text{in}} \end{array}$$

**Figure 8:**
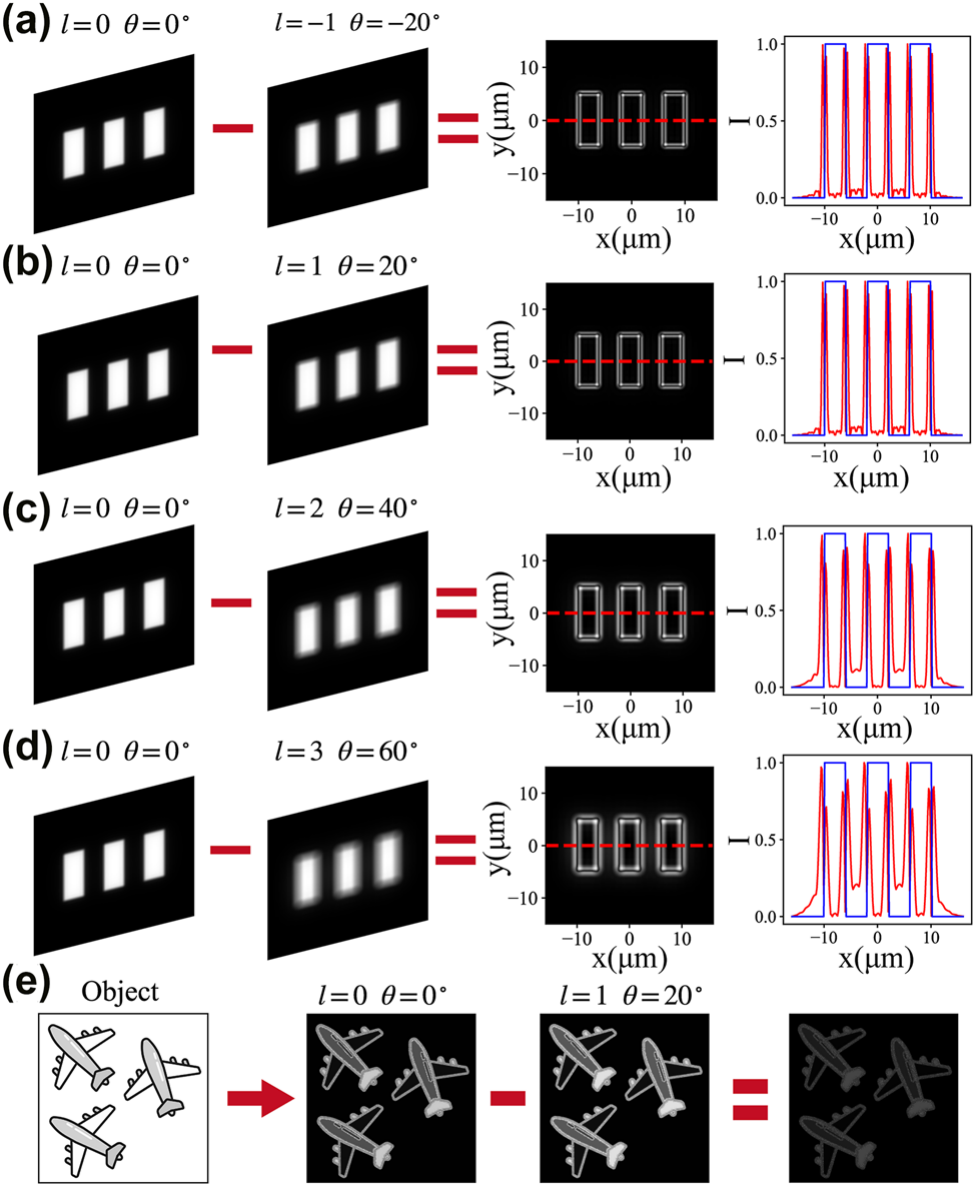
Edge-enhanced imaging results under incoherent illumination using the cascaded metasurfaces. (a)–(d) Imaging at relative rotation angles of −20°, 20°, 40°, and 60°, compared with the image at 0° rotation, along with the corresponding edge-extracted results. Red lines indicate the extracted edges, while blue lines represent the actual sample distribution. (e) Edge extraction results for complex multi-object targets.

Here, the PSF represents the intensity distribution in the image plane, which can be described as the squared magnitude of the amplitude point spread function $\text{PSF}={\left|psf\right|}^{2}$ . The corresponding PSF image under incoherent illumination is shown in Figure 5(b). Unlike the coherent case, it cannot produce interference-based cancellation during the convolution process. Consequently, an incoherent imaging system is linear in terms of intensity, meaning the incoherent PSF is strictly non-negative, and edge-enhanced images cannot be obtained directly under incoherent illumination. To further analyze this behavior, we examine the system in the frequency domain. As detailed in Supplementary Section S4, the Fourier transform of the PSF corresponds to the optical transfer function (OTF). As shown in Figure S4, the OTFs corresponding to topological charges *l* = 1 ∼ 5 do not exhibit high-pass filtering characteristics, and therefore cannot directly realize edge extraction. However, when the OTFs of *l* = 1 ∼ 5 are subtracted from that of *l* = 0, the resulting synthetic OTF redistributes the spatial frequencies of the output image, effectively exhibiting a high-pass filtering response. Moreover, lower-order topological charges remove more high-frequency components, leading to smoother edge transitions. This allows feature extraction and edge detection to be achieved using two acquisitions followed by a single digital subtraction. When the relative rotation angle of the cascaded metasurfaces is 0°, the PSF converges to a typical Airy pattern. After rotating the metasurfaces, the PSF forms a ring due to the additional orbital angular momentum imposed by the spiral phase. Under identical acquisition conditions, the images collected with these two configurations can be digitally subtracted, yielding an optical transfer function (OTF) with weaker intensity at the center and enhanced intensity at the edges, effectively implementing a high-pass filtering response. Convolution of the object with this OTF suppresses low-frequency information at the spot center while preserving high-frequency content, thus achieving edge-enhanced imaging. In practice, we acquired images at relative rotation angles of −20°, 20°, 40°, and 60° and obtained the edge-enhanced image by taking the absolute difference between each rotated image and the 0° reference. The USAF 1951 resolution target, Group 7, Element 1, consisting of three rectangular slits, was selected as the amplitude object for demonstration (Figure 8(a)–(d)). Edge information appears in the valleys between the dual peaks after subtraction. For rotation angles with equal magnitude but opposite directions, the edge patterns are nearly identical (Figure 8(a) and (b)). As the topological charge increases, the edges become slightly blurred and the contrast decreases, since a spiral phase with a lower topological charge more effectively suppresses low-frequency components [46], resulting in higher spatial resolution for edge detection. In this imaging mode, the minimum resolvable line corresponds to Group 7, Element 1, with a spatial frequency of 128 lp/mm and a line width of 4 μm. Moreover, the difference in the resolvable spatial frequencies under incoherent and coherent illumination originates from the intrinsic distinction in their edge-formation mechanisms. As shown in Supplementary Section S10, under incoherent illumination, the extracted *l* = 5 edge appears on one side of the dual-peak response, leading to an effective resolvable feature size of approximately four times the full width at half maximum (∼4FWHM). In contrast, in the coherent system (Supplementary Section S8), the *l* = 5 edge corresponds to a single-peak response, and the minimum resolvable feature size is approximately 2FWHM. This fundamental difference results in a significantly lower practically resolvable spatial frequency in the incoherent case (128 lp/mm) compared with the coherent system (228 lp/mm). The dual-peak nature of the incoherent edge response inevitably broadens the effective edge width and reduces the achievable spatial frequency, whereas the coherent single-peak response preserves a narrower edge profile and enables the resolution of finer features.

We further demonstrate the capability of the proposed device for complex multi-object recognition, which is critical for applications such as autonomous driving. Three airplane targets oriented in different directions were selected, and the corresponding imaging results are shown in Figure 8(e). The cascaded metasurfaces successfully enhances the edges of key structural features, including the wings, fuselage, and tail of each aircraft. This indicates that the device can serve as an optical pre-processing element for optical computing, enabling rapid and efficient extraction of fundamental image features. In Supplementary Section S6, we also perform global normalization for the edge-extraction results from *l* = 1–5. Unlike the coherent case, the peak intensity of the airplane image increases monotonically with the topological charge. The maximum peak value at *l* = 1 reaches only about 60 % of that at *l* = 5. This trend is opposite to that observed in the coherent imaging system. As shown in Figure S4, when the topological charge decreases, more low-frequency components of the OTF are suppressed, which inevitably results in a reduction of overall signal intensity. Conversely, increasing the topological charge improves the signal strength but comes at the cost of reduced system resolution, as discussed in detail in Supplementary Section S8.

## 4 Conclusions

In summary, we propose and demonstrate a Moiré metasurfaces structure compatible with both coherent and incoherent optical systems. By rotating one metasurface relative to the other, the device can generate vortex beams with tunable topological charges from −5 to 5 as well as an Airy disk. This capability enables dynamic switching between bright-field imaging and isotropic edge-enhanced imaging of complex multi-object targets, applicable to both amplitude and phase objects. Under coherent illumination, the edge-detection spatial frequency reaches 228 lp/mm with a line width of 2.19 μm, while under incoherent illumination, it reaches 128 lp/mm with a line width of 4 μm, confirming the device’s performance in diverse imaging and feature-recognition scenarios. The relatively lower performance under incoherent illumination originates from the intrinsic low-pass nature of the incoherent optical transfer function (OTF), which peaks at the center spatial frequency and therefore cannot directly implement high-pass differentiation required for edge extraction. As a result, current incoherent imaging schemes generally rely on acquiring two measurements followed by digital subtraction, or employ optical multiplexing methods such as wavelength or polarization multiplexing to achieve differential imaging. Although such approaches involve partial computation, they still offer significant advantages over conventional digital processing, with the computational cost reduced proportionally to the square of the image pixel number [44]. To further advance toward fully all-optical incoherent edge detection, future designs could integrate the digital subtraction functionality directly into the metasurface itself. It is worth noting that the intrinsic differences in the imaging mechanisms of coherent and incoherent systems lead to fundamentally different behaviors in edge extraction. In the coherent system, increasing the topological charge enhances the edge-resolving capability, enabling the discrimination of progressively narrower line widths. However, this improvement comes at the expense of greater energy loss due to stronger suppression of low- and mid-frequency components. In contrast, in the incoherent system, higher orders enhance the overall image visibility, but this improvement is accompanied by a degradation of fine details and spatial resolution. In addition, to address potential spacing variations between the cascaded metasurfaces, we develop a phase-iteration algorithm based on plane-wave angular spectrum propagation. With this algorithm, the metasurface maintains robust performance over a large spacing range of 0.9–1.1 mm, achieving an average focusing efficiency of 82 % and vortex beam purity up to 99 %, while exhibiting negligible variations in both FWHM and PTP. Beyond its compactness and tunability, the multifunctionality of the device enhances its adaptability and generality across different applications. Compared with conventional digital image processing, it also offers advantages in parallel processing and efficiency. Therefore, the proposed metasurface device holds promise for broad applications, including autonomous driving, computer vision, and biometric recognition, while advancing the development of ultrathin optical devices.

## Supplementary Material

Supplementary Material Details
