# Supplementary material for: Large-gap cascaded Moiré metasurfaces enabling switchable bright-field and phase-contrast imaging compatible with coherent and incoherent light
Source: Nanophotonics. 2025 Dec 2;14(27):5495–509. doi: 10.1515/nanoph-2025-0494 (PMC12717897; doi:10.1515/nanoph-2025-0494)
Supplement: Supplementary file 1 — Supplementary Material Details [file j_nanoph-2025-0494_suppl_001.pdf]

Supplementary Material

Large-Gap Cascaded Moiré Metasurfaces Enabling Switchable Bright-Field and Phase-Contrast Imaging Compatible with Coherent and Incoherent Light

Yiyi Li[1], Wangzhe Zhou[1], Yuqing Zhang[2], Xiaoyan Huang[1], Yutai Chen[1], Man Yuan[1], Junbo Yang[1]*

[1]*College of Science, National University of Defense Technology, Changsha 410073, China*
[2]*School of Physical Science and Technology, Southwest University, Chongqing 400715, China*
**yangjunbo@nudt.edu.cn*

S1. Nanofabrication Processes of Metasurfaces

The fabrication process involves electron-beam lithography and reactive ion etching, as illustrated in Figure S1. A 700 nm-thick titanium dioxide (TiO_2) layer is first deposited on a SiO_2 substrate. A photoresist layer is then spin-coated onto the TiO_2 surface and patterned using an electron-beam lithography (EBL) system. After exposure, a chromium (Cr) layer is deposited to serve as an etching mask, followed by a lift-off process to define the pattern. Subsequently, the structure is transferred into the TiO_2 layer via reactive ion etching (RIE). Finally, the remaining Cr mask is removed, completing the fabrication of the Moiré metasurfaces.

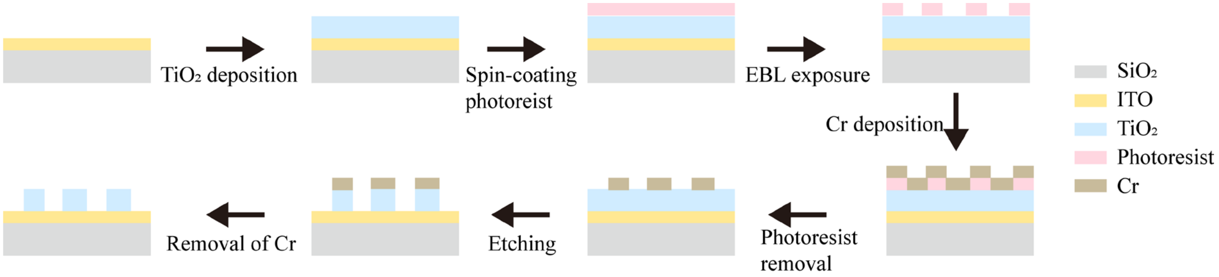

Figure S1. Schematic illustration of the potential fabrication steps for the Moiré metasurfaces.

S2 Analysis of Misalignment

Misalignment is a critical factor influencing the imaging quality of the Moiré metasurfaces system. The displacement between the two metasurfaces along the direction perpendicular to the optical axis (*z*-axis) can be decomposed into a lateral misalignment along the x-direction and a longitudinal misalignment along the y-direction, denoted as Δx and Δy, respectively, as illustrated in Figure S2(a). When Δx is set to 20 and 40 periods, the relative rotation between the two metasurfaces is varied from 0° to 100° in 20° increments. The corresponding PSF intensity distributions in the *x–y* plane are shown in Figure S2(b). Under misaligned conditions, the system continues to switch between a focused spot and vortex beams with topological charges ranging from 1 to 5 as the rotation angle increases from 0° to 100°. However, as the degree of misalignment increases, the vortex beams become increasingly distorted, and the nonuniformity becomes more pronounced at higher topological charges. This indicates that vortex beams are more sensitive to phase errors than focused spots. To quantitatively assess the influence of misalignment, simulations are performed for both lateral and longitudinal cases to obtain the corresponding modulation transfer function (MTF) curves, as presented in Figures S2(c) and S2(d). The cutoff frequency remains nearly unchanged for small misalignments. When the displacement is within 20 periods, both lateral and longitudinal MTFs remain stable, indicating good system robustness. However, when the misalignment exceeds 80 periods, the MTF curves drop significantly, and the spatial frequency corresponding to MTF = 0.5 decreases to approximately 300 mm^{-1}. Moreover, longitudinal misalignment exhibits greater sensitivity compared to lateral misalignment. The relationship between misalignment and system efficiency is illustrated in Figure S2(e). As the displacement increases, the overall efficiency of the system decreases for both lateral and longitudinal misalignments. When the displacement reaches 160 periods, the efficiency is reduced by nearly half compared to the perfectly aligned condition. These results demonstrate that misalignment exerts a non-negligible impact on the performance of the Moiré metasurfaces system, emphasizing the necessity for precise control of lateral and longitudinal alignment errors during fabrication and assembly.

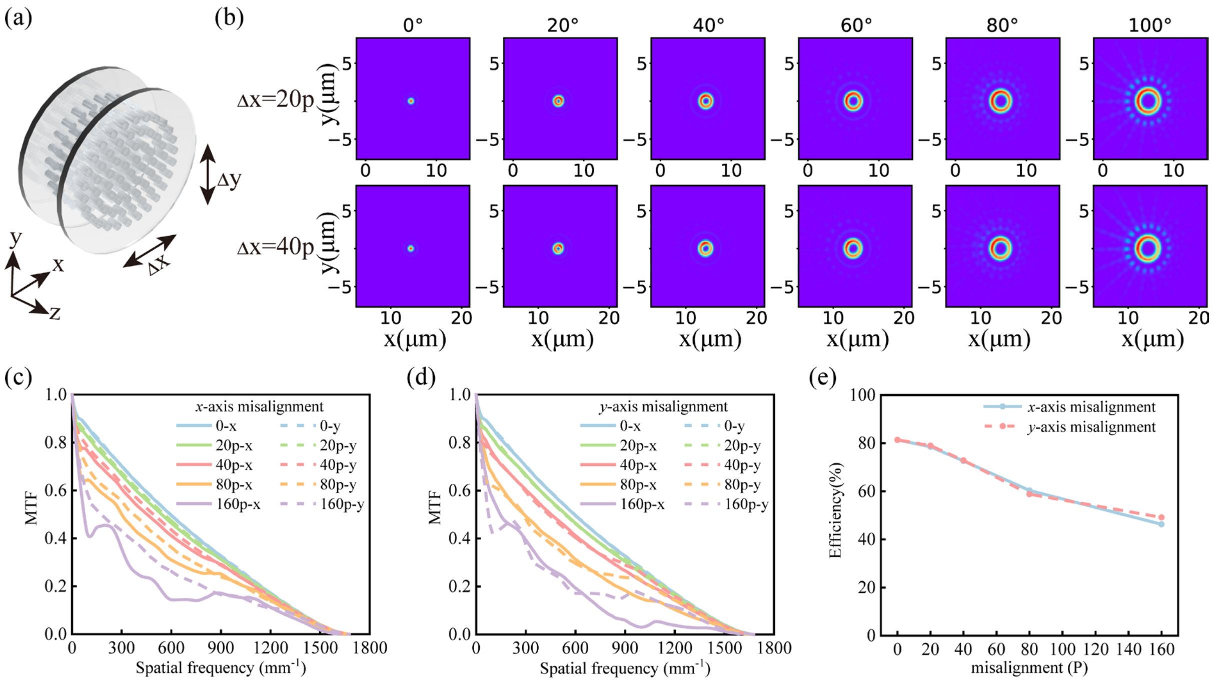

Figure S2. (a) Schematic of lateral (Δ*x*) and longitudinal (Δ*y*) misalignment between the two Moiré metasurfaces. (b) Simulated PSF intensity distributions in the *x*–*y* plane for Δ*x* = 20p and 40p under relative rotation angles of 0°, 20°, 40°, 60°, 80°, and 100°. (c) and (d) MTF curves corresponding to lateral and longitudinal misalignments, respectively. (e) Variation of system efficiency as a function of lateral and longitudinal misalignment.

S3. Analysis of Rotational Accuracy

Rotational accuracy is another critical factor affecting the imaging quality of the Moiré metasurfaces system. The theoretical relative rotation angle between the two metasurfaces is denoted as θ, while the actual rotation angle is $\theta + \Delta\theta$, as illustrated in Figure S3(a). Simulations are performed for rotational deviations $\Delta\theta$ of 0.5° and 1°, and the corresponding PSF intensity distributions in the *x*–*y* plane are shown in Figure S3(d). When the rotation angle θ is set to 0°, 20°, 40°, 60°, 80°, and 100°, the system successively switches between a focused spot and vortex beams with topological charges ranging from 1 to 5. As the rotational deviation increases, the vortex beams become increasingly nonuniform, indicating that they are more sensitive to rotation errors than focused spots. Furthermore, the nonuniformity becomes more pronounced for vortex beams with higher topological charges. To quantitatively evaluate the influence of rotational accuracy, simulations are carried out for relative rotation deviations $\Delta\theta$ of 0°, 0.5°, 1°, 2°, 4°, and 8°, and the corresponding MTF curves are presented in Figure S3(b). When the rotational deviation is within 1°, the MTF remains nearly unchanged, suggesting stable system performance. However, as the rotation deviation continues to increase, the MTF curves decline significantly. When $\Delta\theta$ reaches 4°, the spatial frequency at MTF = 0.5 decreases to approximately 300 mm^{-1}. The relationship between rotational accuracy and system efficiency is shown in Figure S3(c). The efficiency decreases gradually as $\Delta\theta$ increases, while remaining nearly constant for deviations smaller than 1°. At present, studies on the rotational accuracy of Moiré metasurfaces remain limited. The results presented here provide valuable insights and guidance for future research aimed at improving rotational alignment and system stability in Moiré metasurfaces-based optical systems.

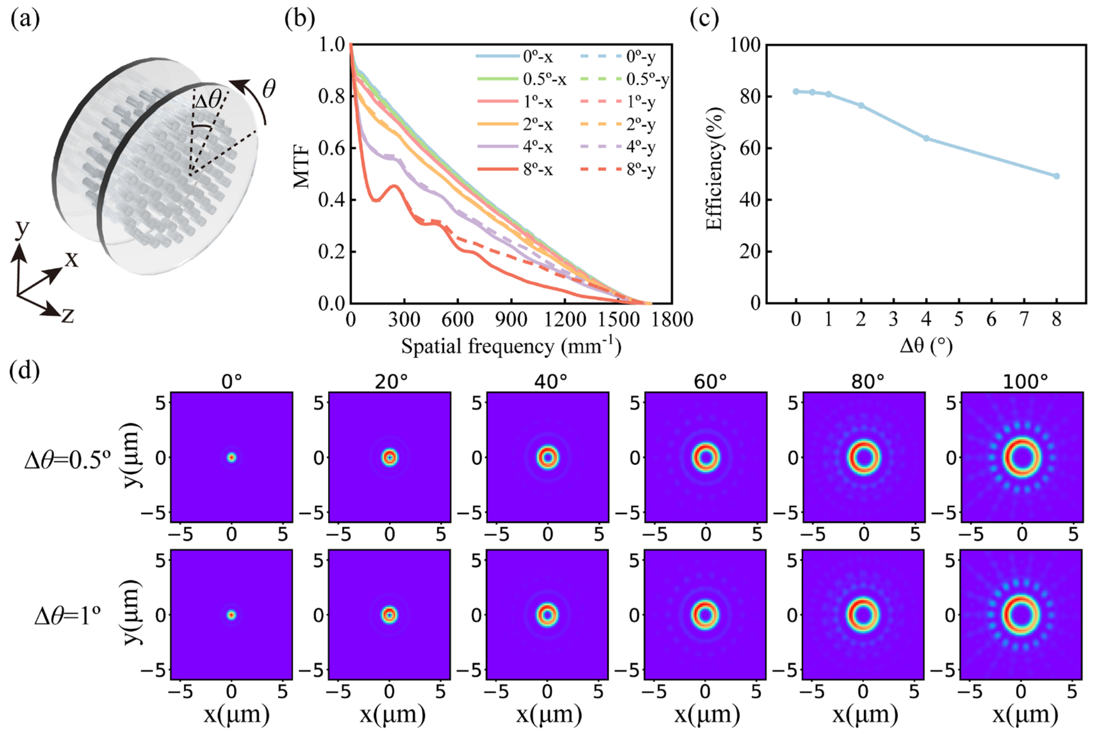

Figure S3. (a) Schematic illustration of the theoretical rotation angle θ and the actual rotation angle $\theta + \Delta\theta$ between the two Moiré metasurfaces. (b) MTF curves corresponding to different rotational deviations $\Delta\theta$. (c) Variation of system efficiency as a function of rotational accuracy. (d) Simulated PSF intensity distributions in the x–y plane for $\Delta\theta = 0.5°$ and 1°.

S4. Performance Analysis of the Incoherent System

By taking the Fourier transform of Eq. (9), the relationship in the frequency domain can be expressed as:

$$I_{out}(k) = \mathrm{T}(k) I_{in}(k) \quad (S1)$$

where $I_{out}(k)$ and $I_{in}(k)$ are the output and input images in the frequency domain, respectively, and $\mathrm{T}(k)$ is the optical transfer function (OTF), given by $\mathrm{T}(k) = \mathcal{F}(PSF)$. The OTFs corresponding to vortex beams with different topological charges are shown in Fig. S4. The synthetic OTFs are obtained by subtracting the OTFs of l = 1~5 from that of l = 0.

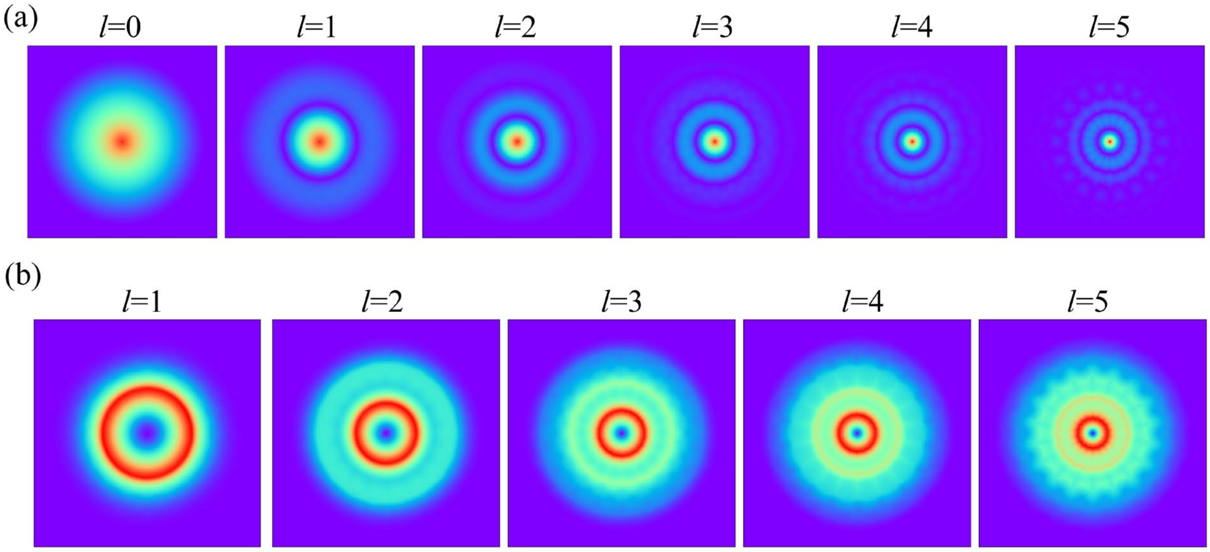

Figure S4. (a) OTFs corresponding to vortex beams with different topological charges l = 1~5. (b) Synthetic OTFs obtained by subtracting the OTFs of l = 1~5 from that of l = 0.

S5. Multi-order imaging performance under coherent illumination

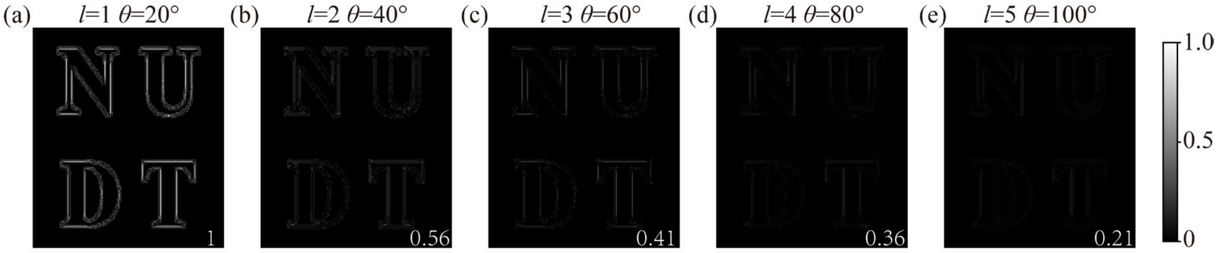

Figure S5. (a–e) Edge-extracted imaging results under coherent illumination for topological charges l = 1~5. The number in the bottom right corner is the maximum intensity value for each image after global normalization.

S6. Multi-order imaging performance under incoherent illumination

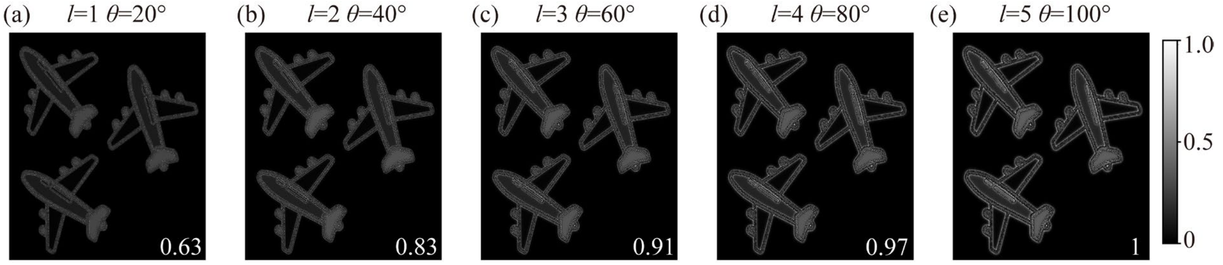

Figure S6. (a-e) Edge-extracted imaging results under incoherent illumination for topological charges l = 1~5. The number in the bottom right corner is the maximum intensity value for each image after global normalization.

S7. Resolution in the Coherent Imaging System

To better examine how multi-order edge extraction varies with spatial frequency, we added single-slit objects with line widths ranging from 1 μm to 16 μm and evaluated their edge-extraction performance for topological charges from l = 1 to 5. The system resolution was quantified by calculating the FWHM of the extracted edges, corresponding to the minimum resolvable feature size. As shown in Fig. S7, when the topological charge is odd, the extracted edges appear at the peak positions; when the charge is even, the edges appear in the valley between two peaks, accompanied by multiple secondary peaks on both sides. Figure S8 shows cross-sectional profiles along the dashed lines in Fig. S7, where the red curves denote the object and the blue curves represent the imaging results. Figure S8(e) shows that for l = 1, 3, 5 the FWHM values of a single slit are 0.849 μm, 0.697 μm, and 0.517 μm, respectively, indicating progressively improved resolution. For a fixed topological charge, the FWHM of the extracted edge remains nearly unchanged as the slit width decreases. Combined with Fig. S8(b), it can be seen that the minimum resolvable feature size for all odd topological charges is 2 μm, where the two slits are still completely separated. When the slit width is further reduced to 1 μm, both the localization of the edges and the enhancement of the secondary peaks degrade for l = 3 and 5, causing the extracted edges in Fig. S7 to deviate from the true object contours.

For l = 4, the interior of the slit is not fully suppressed. Compared with l = 2, the double-line edge feature becomes weaker because one of the two peaks is significantly reduced, making the result resemble that of a single slit; additionally, higher order enhances the corner-like features. When the slit width is 8 μm, as shown in Fig. S8(d), the edges for l = 2 are resolvable, but the slit interior is not fully removed due to the influence of side lobes. When the slit is reduced to 4 μm (Fig. S8(c)), the influence of the side lobes increases further. Although edge extraction remains resolvable, the contrast decreases and the edge quality degrades. When the slit width reaches 2 μm (Fig. S8(b)), the slit is exactly at the resolution limit, where the average FWHM of the two main peaks is 0.558 μm, corresponding to a minimum slit width of approximately four times the FWHM, in agreement with theory. Under the same condition, the average FWHM for l = 4 is 0.495 μm; the slit remains resolvable, but compared with l = 2, the outer-side peaks are further strengthened, meaning the extracted edges tend to emphasize regions slightly outside the true boundaries.

At very high spatial frequencies, the influence of secondary peaks becomes dominant and may even exceed the main-peak intensity, as seen in the case of l = 5 in Fig. S8(b). Excessive filtering of low- and mid-frequency components further amplifies the noise-like high-frequency information.

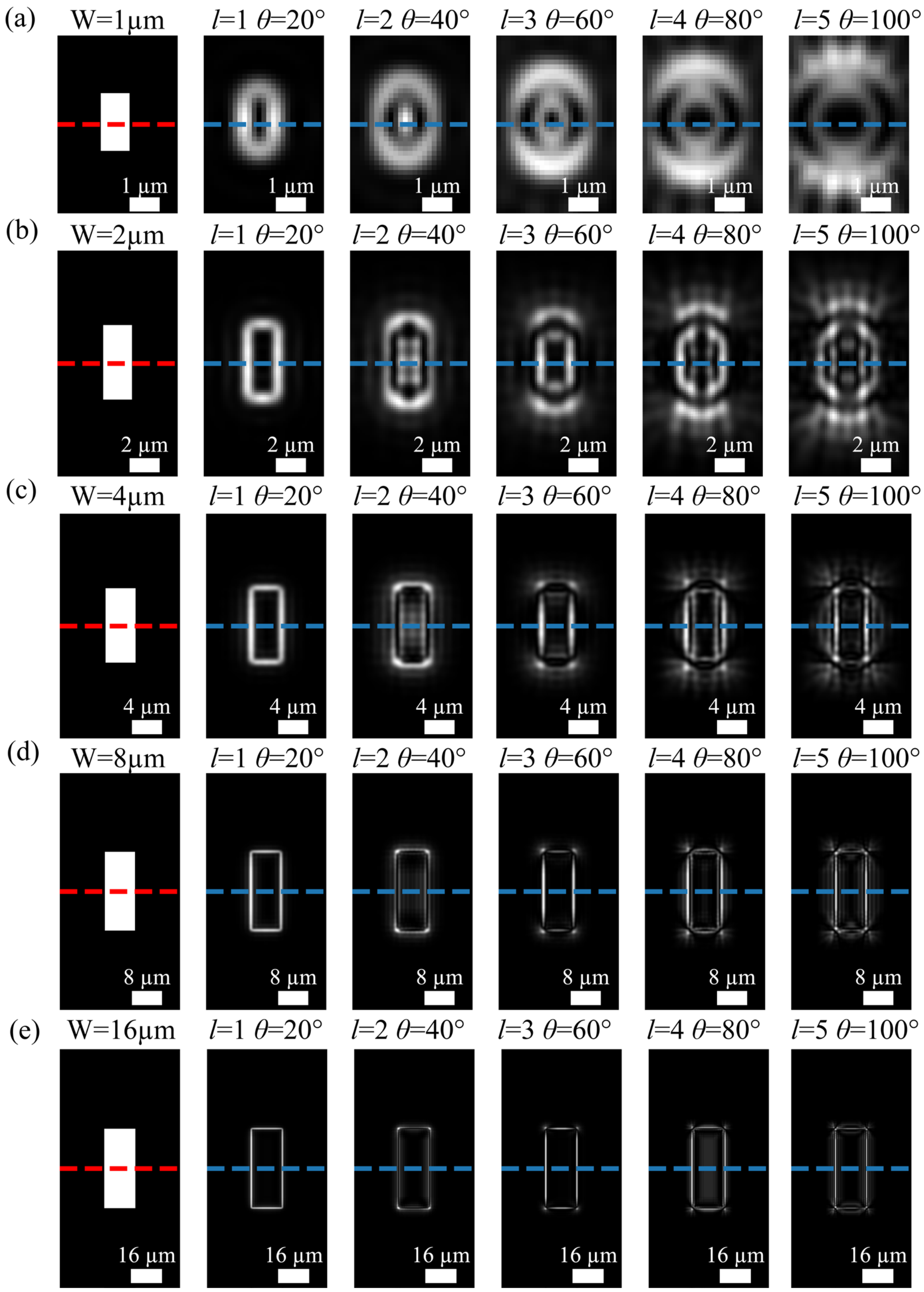

Figure S7. Under coherent illumination, (a–e) show the object images and the corresponding edge-extraction results for l = 1~5, with single-slit widths of 1, 2, 4, 8, and 16 μm.

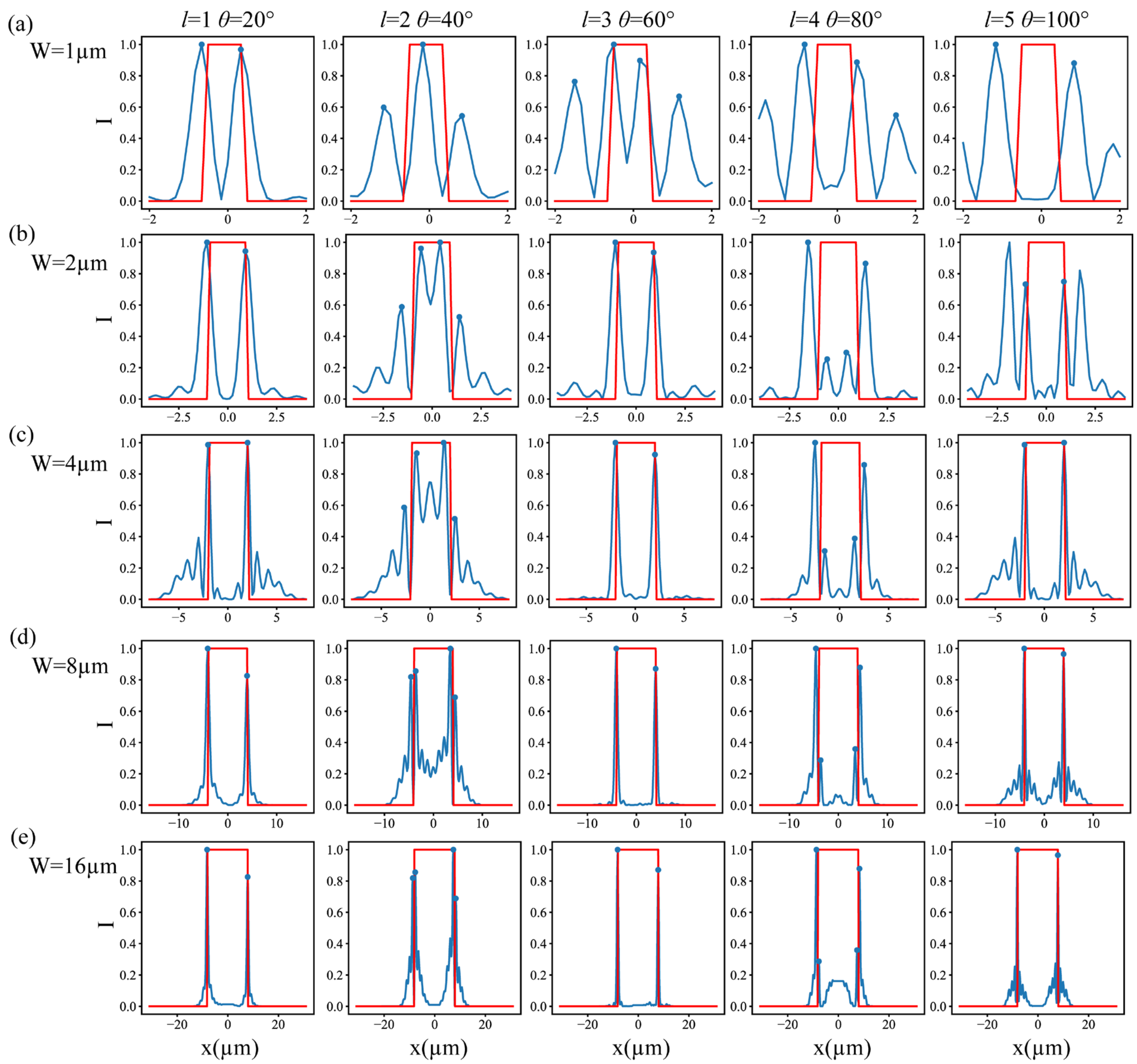

Figure S8. Under coherent illumination, (a–e) show the 1D cross-sectional object profiles and the corresponding edge-extraction profiles for 1~5 , corresponding to Fig. S7, for slit widths of 1, 2, 4, 8, and 16 μm.

S8. Resolution in the Incoherent Imaging System

Similarly, to investigate how the resolution of the incoherent imaging system varies with the topological charge, single-slit objects with widths of 2, 4, and 8 μm were examined for edge extraction under l = 1 to 5, as shown in Fig. S9. Unlike the coherent case, the extracted edges in the incoherent system appear at the valley between two peaks, and no additional side lobes are present. Figure S10 shows the cross-sectional profiles along the dashed lines in Fig. S9, where the red curves denote the object and the blue curves represent the imaging results. As shown in Fig. S10(c), the average FWHM values for l = 1~5 are 0.43 μm, 0.58 μm, 0.77 μm, 0.96 μm, and 1.17 μm, respectively. Therefore, as the topological charge increases, the feature size of the bright peak in the intensity distribution gradually broadens, as observed in Fig. S9—opposite to the trend in the coherent system. As established in Section S4, a smaller topological charge filters out more low-frequency information, enhancing edges and sharpening fine details. When the slit width is 4 μm, as shown in Fig. S10(b), the slit corresponding to l = 5 is exactly at the resolution limit, where the minimum resolvable slit width agrees with approximately four times the FWHM, consistent with both theory and simulation. When the slit width is further reduced to 2 μm, the slit corresponding to l = 2 reaches the resolution limit. Increasing the slit width beyond this point leads to inaccurate edge localization or even the disappearance of the characteristic double-peak edge feature, as shown in Figs. S9(a) and S10(a).

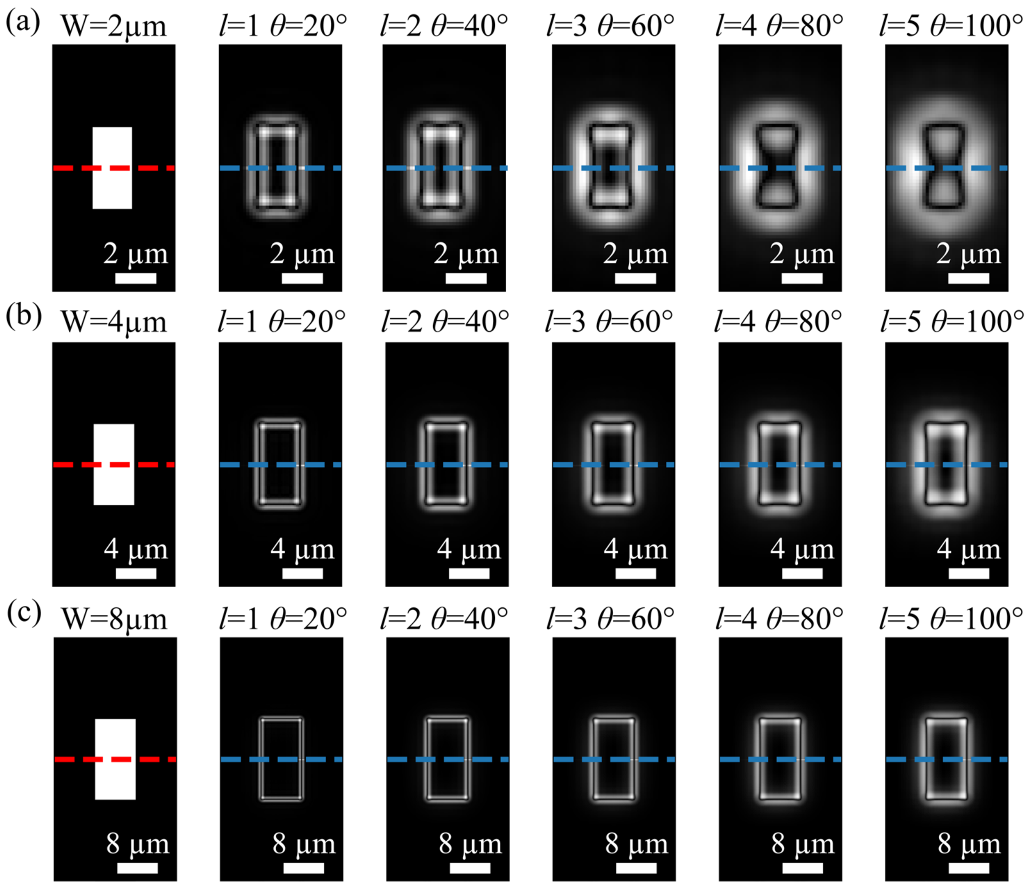

Figure S9. Under incoherent illumination, (a–c) show the object images and the corresponding edge-extraction results for l=1~5 , with single-slit widths of 2, 4, and 8 μm, respectively.

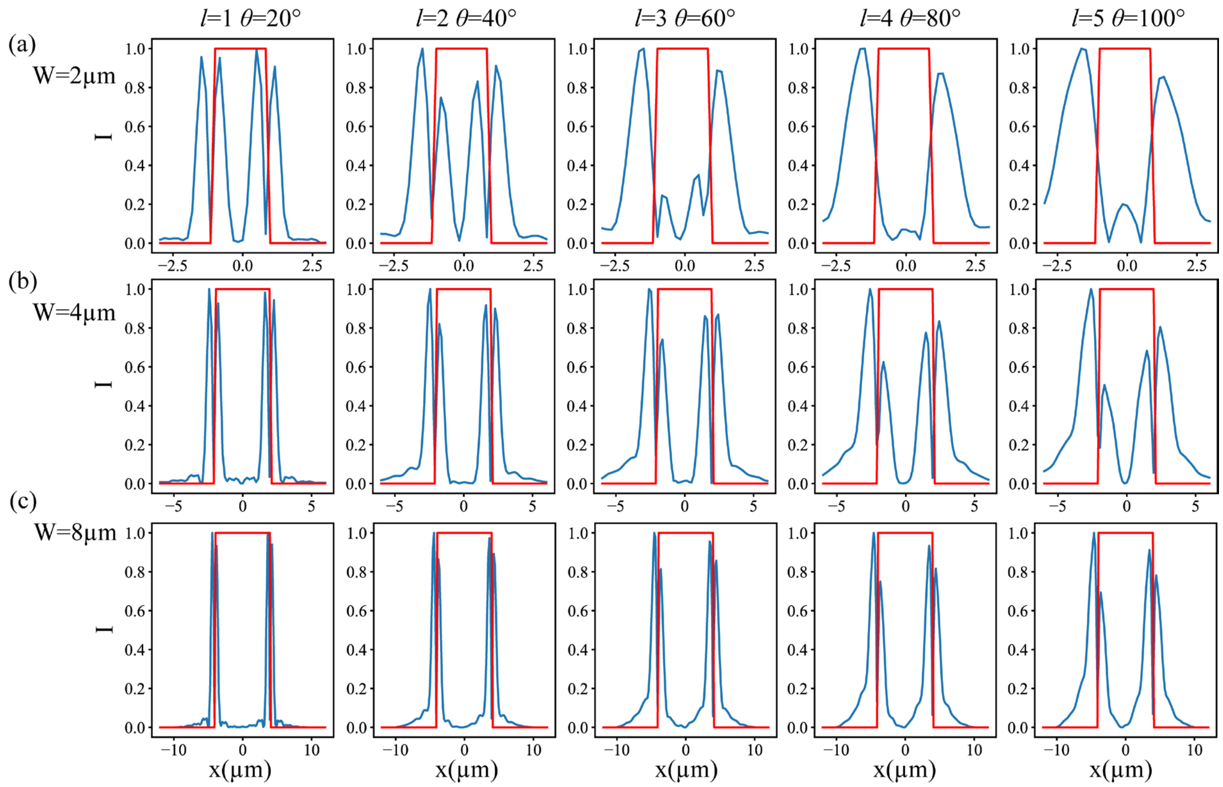

Figure S10. Under incoherent illumination, (a–c) show the 1D cross-sectional object profiles and the corresponding edge-extraction profiles for *l*=1~5, corresponding to Fig. S9, for slit widths of 2, 4, and 8 μm.
